# Electrochemical Redox Refrigeration

**DOI:** 10.1038/s41598-019-50118-y

**Published:** 2019-09-26

**Authors:** Ian S. McKay, Larissa Y. Kunz, Arun Majumdar

**Affiliations:** 1 Department of Chemical Engineering, Stanford University, Stanford, CA 94305 USA; 2 Department of Mechanical Engineering, Stanford University, Stanford, CA 94305 USA; 3 Stanford Precourt Institute for Energy, Stanford, CA 94305 USA

**Keywords:** Electrochemistry, Thermoelectric devices and materials

## Abstract

The high conformational entropy change of the Fe(CN)_6_^3−/4−^ redox reaction can be used as the basis for a compact electrochemical refrigerator. This device is comparable to a liquid version of a Peltier cooler, with two distinct advantages: (1) the entropy change per carrier (1.5 mV/K) of the electrochemical refrigerant is more than 5 times larger than that of state-of-the-art solid thermoelectric materials; and (2) the liquid electrolyte can be advected continuously away from the cooling junction, so that Joule heating in the bulk element does not diminish the delivered cooling effect. In this work, we use infrared microscopy to visualize the thermal aspects of Fe(CN)_6_^3−/4−^ redox, and compare the estimated cooling to calculated values with and without electrolyte flow. While the temperature differences achieved in a single cell are small (~50 mK) and not enhanced by electrolyte flow, the cooling power density (~0.5 W/cm^3^) is high when normalized to the small electrode volume. Non-dimensional figures of merit are proposed to identify electrochemical redox species for maximizing the cooling effect.

## Introduction

Air conditioning and refrigeration are major energy users in any modern economy. In the US, for example, roughly 20% of all electrical power is used by cooling appliances in homes, and 25% is used for climate control in commercial buildings^1^. By 2050, the world population will grow by an additional 2.5B, mostly in urban areas in Asia and Africa that have hot and humid tropical climates^2^. When combined with significant economic growth in these regions, it seems likely that the air conditioning and refrigeration sector will undergo marked growth in the next 10–30 years. If this expansion occurs with today’s cooling technology, it could significantly accelerate global climate change.

It is well known that today’s refrigerants, such as hydrofluorocarbons (HFCs) and hydrochloro-fluorocarbons (HCFCs), have specific global warming potentials about 2000 times that of CO_2_^3^. It has been predicted that because of the global growth of the refrigeration and air conditioning industry, the impact of global warming owing to refrigerant leakage alone could grow to be about 10–40% that of all emitted CO_2_^4^. It is, therefore, imperative that HFCs and HCFCs be phased out in favor of more environmentally friendly alternatives^5^.

A key parameter in the thermodynamics of any cooling cycle is the entropy change, *ΔS*, of the energy carrier. For HFCs, *ΔS* is typically ~1 kJ/kg-K (or ~1 mV/K per carrier)^6^. While the *ΔS* associated with thermoelectric, electrocaloric, magnetocaloric, thermoacoustic, thermoelastic, absorption, and other phase transitions have been studied intensively for cooling purposes^7,8^, no alternative approach has yet provided the reversible and controllable *ΔS* required to be a viable at-scale alternative to vapor compression refrigeration.

One under-studied source of *ΔS* can be found in electrochemical redox reactions. Redox processes are controllable, reversible, can manifest large entropy changes per unit charge (in excess of 2 mV/K over a broad temperature range), and have been harnessed in a wide variety of practical and profitable applications^9^. In thermogalvanic systems, which are electrochemical analogues of solid-state thermoelectric devices, entropy changes inherent in redox reactions are used to couple a flow of ions to a flow of heat for energy harvesting purposes^10^. Recent advances in this concept include both thermally regenerative and continuous electrochemical heat engines, in which forced convection of redox-active fluids allows for decoupling of ionic and thermal transport lengths that may lead to higher efficiencies than are achievable by solid-state thermoelectrics^11,12^. In addition to leveraging the fundamental reaction entropy, thermo-electrochemical systems that leverage the interaction of electrochemical driving forces and other phase transitions have also been deployed successfully^13,14^.

**Figure 1:**
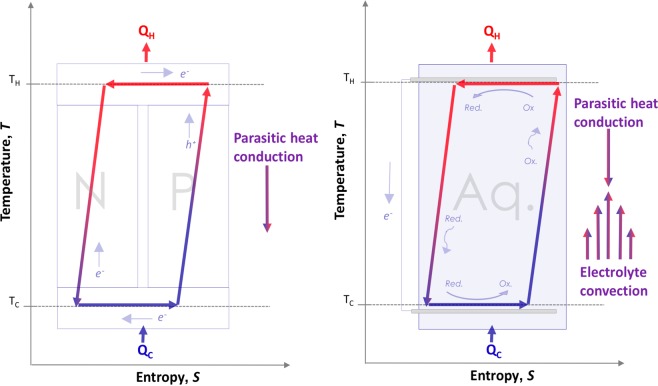
Comparison of idealized Peltier cooler (left) and idealized redox refrigerator (right). Forced advection of joule-heated electrolyte away from the cooling junction in the redox refrigerator may reduce or eliminate parasitic heat losses, enabling higher cooling power.

Despite the interest in energy harvesting, however, work to date on electrochemical refrigeration systems has been largely speculative. Though previous authors have anticipated an electrochemical cooling effect^15^ and even modeled double redox junction refrigeration systems^16^, the effect has not been demonstrated in practice. This omission from the literature is striking because a simple single-junction redox refrigerator has a fundamental advantage over its solid-state equivalent, as illustrated in Fig. 1. Since thermal and electrical conduction are inextricably linked in solid materials, Joule heating from the bulk element necessarily diffuses towards the cooling junction and constitutes a parasitic load. This creates an upper limit on the cooling power of Peltier coolers^17^. By contrast, if a fluid is used instead, the Joule heating can be transported away from the cold junction via advection. In other words, a fluid thermoelectric can actively advect joule-heated electrolyte away from the cooling junction with no penalty to the entropy transport, potentially unlocking higher maximum power. Hence, a fluid-based redox refrigerator can operate without this upper bound in performance.

In this work, we demonstrate the redox cooling effect both in the presence and absence of flow. We use a single-junction redox refrigerator based on the Fe(CN)_6_^3−/4−^ redox couple, which has been shown previously to possesses the requisite low activation energy, high entropy change, and high overall reversibility for effectively coupling thermal and electrical energy flows^18^. When a potential difference is applied across a pair of electrodes immersed in the electrochemical refrigerant, Fe(CN)_6_^4−^ is oxidized to Fe(CN)_6_^3−^ at the anode, producing a cooling effect, while the opposite process occurs at the cathode, rejecting heat. An overall flow of electrolyte from the cooling to the heating electrode prevents the equilibration of the cooling electrode with either the heating electrode or the joule-heated electrolyte. In the cell, the reduction of Fe(CN)_6_^3−^ yields an entropy change per charge *ΔS/F* = −1.5 mV/K, which corresponds to a theoretical maximum cooling power density of *iΔST*_*c*_*/F* for applied current density *i*, Faraday’s number *F*, and cold electrode temperature *T*_*c*_^19^. We explore the effect of electrolyte convection on temperature depression and observe the cooling effect in two configurations. Figures of merit are proposed for redox refrigerants, and a number of conventional redox active fluids from the flow battery community are evaluated as potential alternatives to the Fe(CN)_6_^3−/4−^ redox couple.

Three time scales govern the dynamics of the Fe(CN)_6_^3−/4−^ electrochemical refrigerator. Considering heat conduction, the characteristic time for transport between the electrodes is *τ*_*thermal*_ ≈ *d*^2^*ρC*_*p*_*/κ* for inter-electrode distance *d*, electrolyte density *ρ*, thermal conductivity *κ*, and specific heat *C*_*p*_. Considering heat advection, the characteristic time depends on the flow velocity *v* between the electrodes *τ*_*flow*_ ≈ *d/v*. For stagnant electrolytes, the characteristic timescale of the electrochemical response based on reactant diffusion is *τ*_*sand*_ ≈ *πD*(*C*_*o*_*F/2i*_*initial*_) for reactant diffusivity *D*, concentration *C*_*o*_, and applied current density *i*_*initial*_^20^. To enable continuous cooling, the cooling electrode must be thermally isolated from both Joule heating in the electrolyte and activation losses at the hot electrode. This requires the electrolyte to flow swiftly enough so that *τ*_*flow*_ < *τ*_*thermal*_. For stagnant electrolyte, the duration that cooling can be maintained is expected to be the shorter of *τ*_*thermal*_ and *τ*_*sand*_.

## Methods

**Figure 2:**
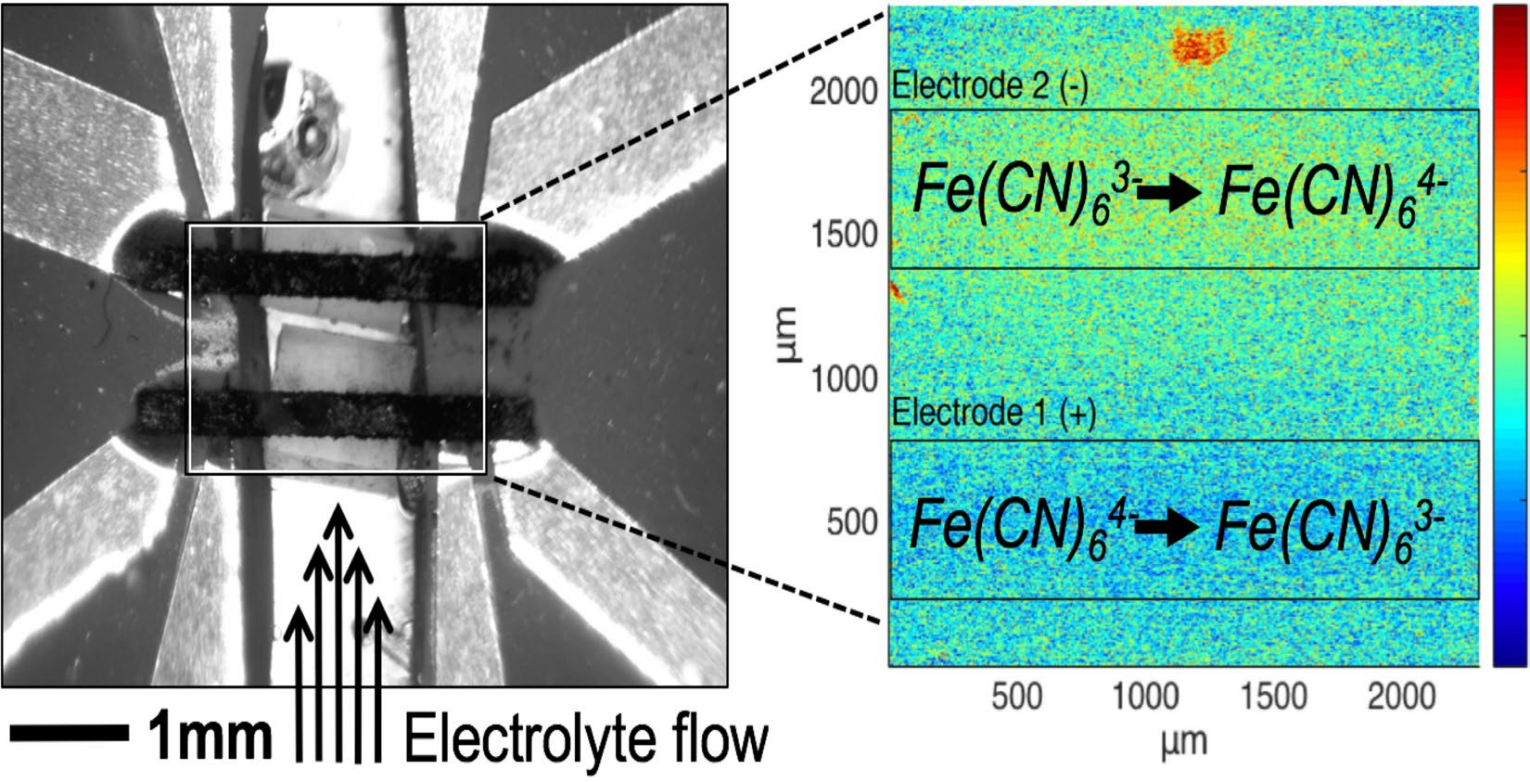
The electrodes and electrolyte flow channel in optical (left) and infrared (right) views.

An infrared microscope (Quantum Focus Instruments, InSb detector) was used to visualize the thermal effects of Fe(CN)_6_^3−/4−^ reduction/oxidation in a custom-made electrochemical flow cell, depicted in Fig. 2. Carbon paper electrodes (SpectraCarb 2050a) were oxidized in air at 200 °C for hydrophilicity and deposited on an infrared-transparent CaF_2_ substrate (ThorLabs). The electrodes were positioned in the flow path of an aqueous 350 mM K_3_Fe(CN)_6_^3−^|350 mM K_3_Fe(CN)_6_^4−^ solution. The electrolyte flow was controlled using a peristaltic pump (Masterflex) and preheated to 50 °C before entering the cell by flowing through a parylene-coated serpentine channel milled into a heated Al block. In addition to heating the sample above the ambient temperature for better infrared signal above room-temperature thermal reflections, this heating allowed the emissivity for each point within the sample to be calibrated. The single-temperature emissivity measurement is made possible by a correlation between one- and two- temperature radiance measurements for a library of materials implemented by the Quantum Focus microscope. More information on the I.R. temperature measurement is available in the S.I. Electrolyte flow velocity was estimated by measuring the volumetric flow rate and correcting for the cross-sectional area of the flow channel housing the electrodes. Contact to the electrodes was established using strips of 12.5 μm Ti foil (GalliumSource), as shown in Fig. 2.

The electrochemical redox of Fe(CN)_6_^3−/4−^ was driven and monitored potentiostatically (Biologic SP-240). Since the opposite electrochemical reactions were run at the oxidation (cooling) and reduction (heating) electrodes, the open-circuit voltage of the cell was 0 V, and the applied voltage is reported as the total overpotential for both reactions *η* = *η*_*red*_ + *η*_*ox*_. No reference electrode was used.

The temperature depression was estimated by integrating the temperature signal over the area of the cooling electrode on the infrared movie that was created from each test run. The total cooling power was estimated using an aggregate heat transfer coefficient, *U* [W/K], between the electrode and its surroundings. To establish *U*, a known electrical current was driven across the cooling electrode to use it as a Joule heater with known power dissipation *P*. By monitoring the resulting electrode temperature rise *ΔT* at each flow setting, the constant of proportionality *U* = *ΔT/P* could be inferred. The *U* measured for each flow rate was then used to estimate the cooling power based on the observed *ΔT* during the cooling experiments.

For stagnant electrolyte tests, both electrodes were loaded with a high activity and high surface area catalyst, which was intended to both decrease activation losses and to increase the emissivity of the electrode for better temperature estimation. The Pt-C catalyst ink consisting of 50 μg/μL HISPEC 40%Pt on high surface area carbon in 3:2:0.1 H_2_O (MilliQ Synergy UV):Isopropanol (Aldrich):Nafion 117 dispersion (Aldrich) was dropcast onto the positive electrodes for a total Pt loading of 0.5 mg/cm^2^. For tests with flowing electrolytes, no catalyst coating was used to improve electrode consistency over lengthy tests. Electrode spacing *d* was also increased from approximately 0.5 mm in the stagnant test to 1.0 mm in the tests with flow.

## Results and Discussion

**Figure 3:**
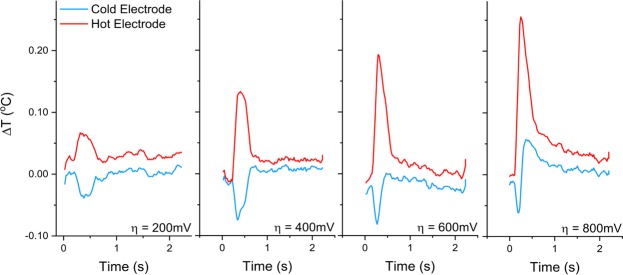
Temperature response of the anode (cold electrode) and cathode (hot electrode) upon application of various overpotentials in stagnant electrolyte. For this configuration τ_sand_ ≈ 0.3 s while τ_thermal_ ≈ 1.4 s, so it is likely that the cooling pulse duration was limited more by ion concentration polarization than by thermal equilibration between the electrodes.

Initial testing was performed in stagnant electrolyte to test the electrochemical and thermal behavior in the absence of flow. The thermal response of both electrodes to 2 seconds of applied voltage is shown in Fig. 3. As expected, the anode experiences a momentary temperature decrease, and the cathode experiences a concomitant increase. Both temperature deviations are of shorter duration than the applied voltage pulse. While the short duration of cooling could be due to either thermal equilibration in the small gap between electrodes or the decreasing reaction current over time due to concentration polarization of the electrolyte (Fig. S2), concentration polarization is likely the larger contributor in this case because *τ*_*sand*_ < *τ*_*thermal*_. As larger driving potentials are applied, the magnitude of the heating effect at the cathode grows faster than the cooling effect at the anode, as expected for less reversible heat pump operation. At very high overpotential, the cooling effect at the anode is overwhelmed by heating due to activation and transport losses even before *τ*_*sand*_, and long before heat can propagate across the inter-electrode space.

**Figure 4:**
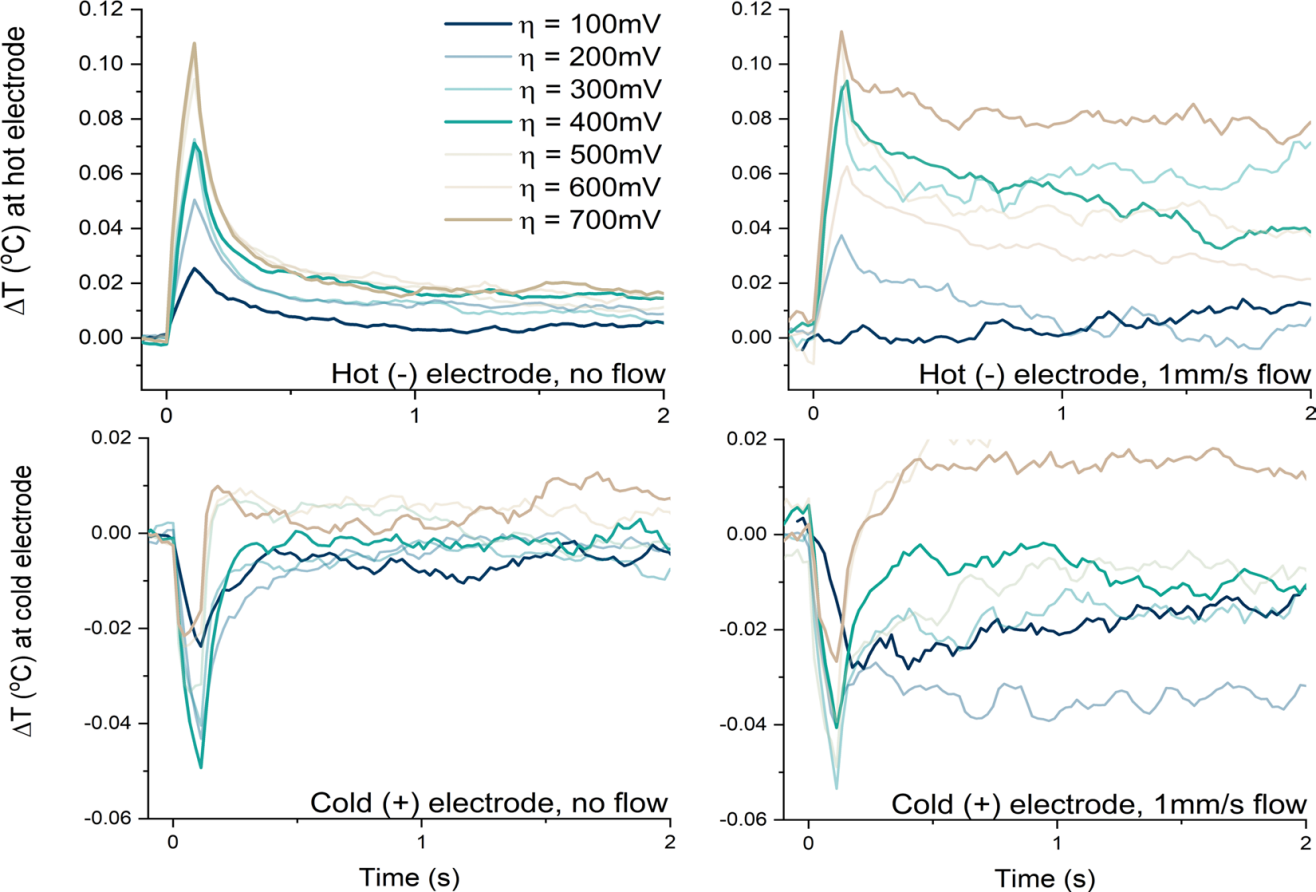
Temperature response of the cold electrode (bottom) and hot electrode (top) upon application of various overpotentials in both stagnant (left panels) and flowing (right panels) electrolyte. Flowing electrolyte enables continuous cooling operation, but it does not significantly change the measured peak temperature depression.

As shown in Fig. 4, electrolyte flow dramatically altered the thermal response of the cell. In these tests, different overpotentials were applied and the temperature response monitored with and without electrolyte flow. The applied current and overpotential were also recorded and were used to compare the estimated to the theoretical cooling power. Both peak and maximum steady-state cooling were achieved at higher overpotential with flowing electrolyte than with stagnant electrolyte. Flowing electrolyte also allowed for some steady-state refrigeration (albeit delivered at lower *η* than the peak value), which was neither expected nor observed in the cell with stagnant electrolyte. However, the magnitude of the peak cooling was not significantly larger with flow than without.

The similarity of the peak cooling values for stagnant and flowing electrolyte is attributable to the scaling of both *i* and *U* with *v*^*1/2*^ (see derivation in SI). In other words, higher flow rates allow for higher cooling power by advection of reactant towards (and Joule heating away from) the cooling electrode but simultaneously allow better heat removal from the cooling electrode. Therefore, electrolyte flow does not appreciably change the transient *ΔT*.

**Figure 5:**
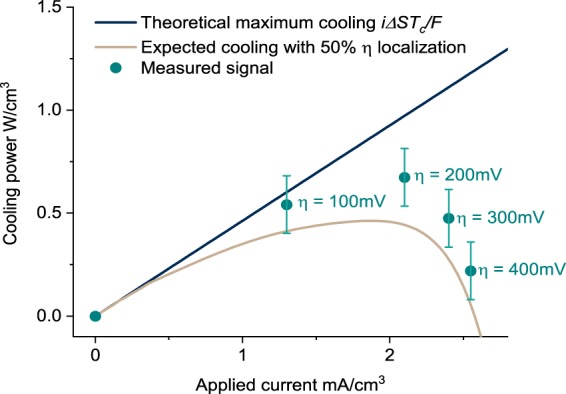
Estimated volumetric cooling power at different driving currents with 1 mm/s electrolyte flow, compared to theoretically achievable cooling power with no losses (black) and with measured irreversibility evenly split between the two electrodes (brown). The solid lines are calculated based on *i* and *η* input to the cell while individual points are based on the thermal signal at the IR microscope. Electrolyte flow from the cold to the hot electrodes allows less than 50% of kinetic losses in the cell to manifest as a temperature rise on the cooling electrode, enabling a more powerful refrigeration effect than would be possible in a cell with stagnant electrolyte.

Comparison of the estimated and theoretical cooling power density with electrolyte flow illustrates a similar dynamic. Since the electrode is porous and has a three-dimensional architecture, it is most useful to present this as a volumetric power density, where the volume is measured using the nominal area and the thickness of the electrode. As shown in Fig. 5, electrolyte flow allows for favorable partitioning of the temperature rise due to the cell’s irreversibility; less than 50% of the total overpotential applied to the cell results in undesired temperature rise at the cooling electrode. For a typical Butler-Volmer transfer coefficient *α*_*b−v*_ ≈ 0.5, the expectation in stagnant electrolyte is that approximately 50% of the applied activation overpotential will manifest as an undesirable temperature increase on the cooling electrode. The anticipated cooling in this case is described by the brown line in Fig. 5. That the measured cooling power was higher than this power indicates the benefit of electrolyte flow in sweeping joule heated electrolyte away from the cooling junction. This effect diminished and the total kinetic losses regressed towards an equal partition at higher currents, as concentration polarization rather than ohmic losses in the electrolyte become dominant^20^. While the observed partitioning of overpotential losses onto the heating electrode could in theory be due to a kinetic asymmetry in the reaction, (i.e. a Butler-Volmer transfer coefficient *α*_*b−v*_ ≠ 0.5), this asymmetry would not continue to scale with higher applied overpotentials and is inconsistent with prior measurements of *α*_*b−v*_ for Fe(CN)_6_^3−/4−^ kinetics^21^.

The combination of CaF_2_- backed electrodes and infrared microscopy used in this experiment proved its worth for visualizing thermal aspects of electrochemical processes. Thermal microscopy provided much better spatial resolution than was required for these measurements, and might prove useful for future authors investigating localized heat generation in different parts of electrochemical cells, or even for parallelized electrocatalytic screening^22^. Temperatures were successfully measured on a variety of porous and nonporous materials, including metallic electrodes (Pt and Pd). While high emissivity is desirable for temperature measurements, it is not always easy to achieve. Our best results for low-emissivity electrodes were achieved by spin-coating a thin layer of photoresist as an emitter layer directly on the infrared-transparent substrate prior to electrode deposition. Even better results might be obtainable by adding an emissivity-enhancing dissolved or colloidal species to the electrolyte itself, or by utilizing thinner porous electrodes.

A number of steps could have been taken to achieve a greater cooling effect but were forgone in this work for experimental simplicity and ease of visualizing results. These include tighter electrode spacing for lower total ohmic losses^16^, a supporting electrolyte for greater ionic conductivity^20^, high surface-area catalyst loading, flow-through rather than flow-past electrode configuration, a multi-stage design (Fig. S3), and a much higher flow rate to eliminate concentration polarization on the cooling electrode. The relatively sluggish flow rates used in this work reflect the goal of elucidating the effect of flow, rather than leading to the maximum possible cooling power. The flow-past electrode configuration was similarly chosen to facilitate lower-noise IR thermography; extensive work in the flow battery community suggests that a flow-through configuration would in fact better reduce concentration polarization^23^. Additionally, while thermodynamic calculations indicate that cooling can be achieved up until a product/reactant concentration *C*_*oxidized*_*/C*_*reduced*_ = *e*^*−αF/R*^ (see derivation in SI), the use of a reference electrode could aid future researchers in localizing overpotential and thus in formulating the ideal ratio of reduced and oxidize species in the refrigerant. We expect that these measures will be taken in future work.

An important aspect of optimizing future electrochemical refrigeration systems will be materials design. While the Fe(CN)_6_^3−/4−^ redox couple was chosen in this work based on its track record of use in thermogalvanic systems, there is a wide parameter space open for electrochemical refrigerants with the right combination of high standard entropies of reduction *ΔS*, low activation barrier *E*_*a*_ for reduction (if *α* > 0) or oxidation (if *α* < 0), low specific heat *C*_*p*_, and a high capacity of entropy carriers *C* (in mol/kg), which equates to high total solubility for dissolved species. These properties are the primary determinants of the achievable cooling effect *ΔT*_*real*_ in a system in which heating due to concentration polarization and ohmic losses in the electrolyte are managed by forced convection.

1 $$\Delta {T}_{real}=\frac{[\Delta S(\frac{J}{mol\cdot K})T(K)-{E}_{a}(\frac{J}{mol})]\times C(\frac{mol}{kg})}{{c}_{p}(\frac{J}{kg\cdot K})}$$

Based on a Buckingham Pi analysis, we propose the following dimensionless figures of merit for electrochemical refrigerants:

2 $$Y=\frac{\Delta S(\frac{J}{mol\cdot K})\cdot C(\frac{mol}{kg})}{{c}_{p}(\frac{J}{kg\cdot K})}$$

and

3 $${Q}_{g/b}=\frac{\Delta S(\frac{J}{mol\cdot K})T(K)}{{E}_{a}(\frac{J}{mol})}$$

*Y* gives the ratio of redox cooling potential to sensible heat energy stored in the refrigerant and is also described in the literature on electrochemical energy harvesting^11^. *Y* expresses the thermodynamic reality; *YT* = *ΔT*_*max*,_ the maximum cooling that could be achieved adiabatically by this refrigerant given totally reversible operation and a very long residence time near the electrode surface (or in a staged design, see SI). By contrast, *Q*_*g/b*_ expresses the kinetic reality as the ratio between the “good” and “bad” thermal signatures per carrier; only refrigerants with *Q*_*g/b*_ > 1 can demonstrate a cooling effect in practice.

Redox reaction entropy is generally attributed to rearrangements of molecular structure and solvation^24^. Marcus theory, however, correctly predicts that large molecular and solvation rearrangements disfavor electron transfer^20^. As a result, one might expect a fundamental tradeoff between reversibility and reaction entropy that limits *Q*_*g/b*_. Due to solvent effects in particular, larger dissolved molecules should demonstrate generally lower *E*_*a*_ and *ΔS* whereas smaller molecules should demonstrate higher *ΔS* and *E*_*a*_. In this respect, a “happy medium” for *Q*_*g/b*_ might be hard to find. To date, the best *Q*_*g/b*_ values have been found in coordinated metal redox couples, which to some extent avoid this kinetic tradeoff with entropies of reduction that are based more on solvent rearrangement than on reorganization of the redox centers themselves^24^.

Table 1 illustrates a different tradeoff. Pure substances such as water require no solvent by definition and so have high *Y* ratios and correspondingly high *ΔT*_*max*_ relative the species’ thermopower. However, redox of these small molecules requires inner-sphere electron transfer, which tends to proceed slowly^20^, leading to a poor ratio *Q*_*g/b*_. By contrast, many coordinated metal species are only moderately soluble, and the *C*_*p*_ contribution of the excess solvent is reflected in low *ΔT*_*max*._ However, these species undergo comparatively fast single-electron transfer and frequently have high *ΔS* leading to high *Q*_*g/b*_. An important step in the future will be to identify the trick that allows an electrochemical refrigerant to circumvent this apparent tradeoff.

**Table 1:** Properties of potential redox refrigerants.

Redox reaction	α, mV/K	YT = ΔТ_max_ @300K, K	Q_g/b_ @300K
2 H^+^ + 2e^−^ → H_2_ (g)	0.8	307	4.0^9,27^
V^3+^ + e^−^ → V^2+^	1.2–1.9*	36	6.0^28^
V^5+^ + e^−^ → V^4+^	−0.2*	6	2.4^28^
Br_2_(l) + e^−^→ 2Br^−^	0.3*	14	1.7^29^
Fe(CN)_6_^3−^ + e^−^ → Fe(CN)_6_^4−^	−1.5*	10.6	78.4^30^
Fe^3+^ + e^−^ → Fe^2+^	1.1*	36	95.7^31^
Cr^3+^ + e^−^ → Cr^2+^	2.2	7	219.5^9,23,31^
Ce^5+^+e^−^ → Ce^4+^	2.3	13	36.0^9,32^
S_2_^2−^ + 2e^−^ → 2S^2−^	−0.7	9.4	1.3^9,33^

*Q*_*g/b*_ and *YT* = *ΔТ*_*max*_ are listed to illustrate the trade-off between overall cooling capacity, reversibility, and thermopower. Properties of reduction reactions denoted ‘*’ were measured expressly for this work, as described in the SI. Entropy of reduction varies with total concentration for some species. In these cases, *ΔТ*_*max*_ and *Q*_*g/b*_ were calculated based on the concentration yielding the highest *ΔТ*_*max*_. Activation energies *E*_*a*_ were estimated based on literature sources for exchange current density or reaction velocity, as described in the SI.

The redox flow battery community has spent decades screening electroactive species for solubility, stability and reversibility^23^, which can now be leveraged to identify electrochemical refrigerants. Further inspiration can be found in the strikingly similar set of compounds found in biological vascular systems. Hemovanadin (vanadium); hemoerythrin, chlorocruorin, and hemoglobin (iron); and hemocyanins (copper) all point to the enormously tunable redox properties of an appropriately coordinated metal center^25^. Laboratory results suggest that the standard entropy of reduction is perhaps as tunable as the redox potential^24^. The inexhaustive list of Table 1 contains only a few potential liquid-phase refrigerants and completely omits other electrochemical transformations (solid dissolution, hydriding, intercalation, solution-precipitation reactions, redox of slurries, use of non-aqueous media etc.) that may be of great interest in future work.

Interestingly, due to the difference in reduction entropies of the V^4+/5+^ and V^2+/3+^ redox couples, a vanadium redox flow battery can already be considered to be producing a beneficial electrochemical cooling effect during charging. However, due to the high rates at which these batteries are charged, it is unlikely that a net cooling effect is ever observed^23^. A more likely scenario is that the battery is discharged during cool evening hours and recharged during the heat of the day. In this case, the entropy changes align to slightly increase the equilibrium voltage during discharging and to slightly decrease it during recharging. This energy harvesting effect likely results in a small but measurable boost in the batteries’ cycle efficiency. Other mooted grid-scale energy storage systems such as Zn-air, Fe-air, and regenerative hydrogen fuel cells would also benefit from this effect.

It’s instructive to compare the performance limits of a redox refrigerator with its solid-state counterpart, the Peltier cooler. In the case of the Peltier cooler, both the maximum cooling power and *ΔT* are governed by a balance of *ΔS* with thermal (*κ*) and electrical (*σ*) transport, as embodied by the thermoelectric figure of merit *ZT* = *σS*^2^*T/κ*^26^. By contrast, the maximum temperature depression and cooling power of a redox refrigerator are independent of transport phenomena; *ΔT*_*max*_ = *YT* is a thermodynamic tradeoff, while the maximum cooling power in the limit of very high electrolyte flow should be hampered only by the redox reaction activation barrier *E*_*a*_, which does not increase with reaction rate. As a result, the redox refrigerator is perhaps most promising as a means to provide high cooling power densities at small scales. For example, a redox refrigerator with the cold-electrode built into a microchip might deliver a high cooling power to an individual transistor or high-activity region of computer memory. However, other applications may be hampered by the drawbacks of redox refrigerators relative solid-state coolers. These include (i) the low *Y* for known refrigerants mean many coolers in series will be required to produce even moderate *ΔΤ*, (ii) relatively large *E*_*a*_ mean that efficient cooling cannot be delivered even at low reaction rates, and (iii) the requirement of flowing electrolyte for continuous cooling, which increases overhead power draw while merely sweeping away—but not eliminating—the heating due to transport irreversibility. As a result, while very high power densities can be achieved normalized to the small cooling electrode volumes, redox refrigerators are unlikely to provide efficient cooling, high coefficient of performance, or even high power density when normalized to an entire device volume.

## Conclusions

An electrochemical refrigerator based on the Fe(CN)_6_^3−/4−^ redox couple was developed and tested. While refrigeration systems have been demonstrated previously that leverage electrode processes to drive other phase transitions^14^, to our knowledge this is the first experimental demonstration of a refrigeration cycle based on the entropy changes inherent in electrochemical redox reactions. Rather than trying to develop an optimized or scaled-up device, we investigate electrode configurations with and without electrolyte flow. Although the high entropy change of Fe(CN)_6_^3/4−^ redox and the ability to flow the joule-heated electrolyte away from the cooling junction enabled high power densities when normalized to the tiny volume of the cooling electrodes, these high power densities did not translate to either large temperature differences or efficient refrigeration. We found that, at least for this redox couple in this configuration, the benefit of electrolyte flow cannot compensate for the high reaction activation energy and low electrolyte conductivity relative to solid-state thermoelectric devices.

A key advantage of the electrochemical approach over other alternative approaches to refrigeration is that it offers the unique opportunity to leverage the vast knowledge and progress in the area of electrochemical storage (e.g. batteries) and to use it in a novel way for the purpose of cooling. As new redox-active species are discovered or designed with improved reversibility and rate capability, electrochemical refrigeration may prove to be a promising alternative for 21^st^-century climate control.

## Supplementary information

Redox Refrigeration - Supplementary Information

## Data Availability

The datasets generated during and/or analysed during the current study are available from the corresponding author on reasonable request.
