# Supplementary material for: Electrochemical Redox Refrigeration
Source: Sci Rep. 2019 Sep 26;9:13945. doi: 10.1038/s41598-019-50118-y (PMC6763465; doi:10.1038/s41598-019-50118-y)
Supplement: Supplementary file 1 — Redox Refrigeration - Supplementary Information [file 41598_2019_50118_MOESM1_ESM.docx]

**Electrochemical Redox Refrigeration**

Ian S. McKay^1^, Larissa Y. Kunz^1^, Arun Majumdar^2,3*^

^1^Department of Chemical Engineering, ^2^Department of Mechanical Engineering, ^3^Stanford Precourt Institute for Energy, Stanford CA 94305 (USA)

**Supplemental Information**

**Supplemental Information**

**Contents**

- - Deriving the maximum possible reaction extent for effective cooling
  - Comparing the scaling of limiting current density *I_lim_* and heat transfer coefficient *U* with flow velocity *v*
  - Calculating *Q_g/b_* and *ΔT_max_* for a variety of redox couples
  - Ruling out an ionocaloric effect in the Fe(CN)_6_^3-/4-^ system
  - Further information on the infrared electrode temperature measurement

Table S1

Figures S1 – S7

**Deriving the maximum possible reaction extent for effective cooling**

The entropy change of a redox process with fundamentally positive conformational entropy change *ΔS_r_* will decrease to zero at high reaction extents when the configurational entropy change of further reaction *ΔS_c_* becomes equal in magnitude and opposite in sign.


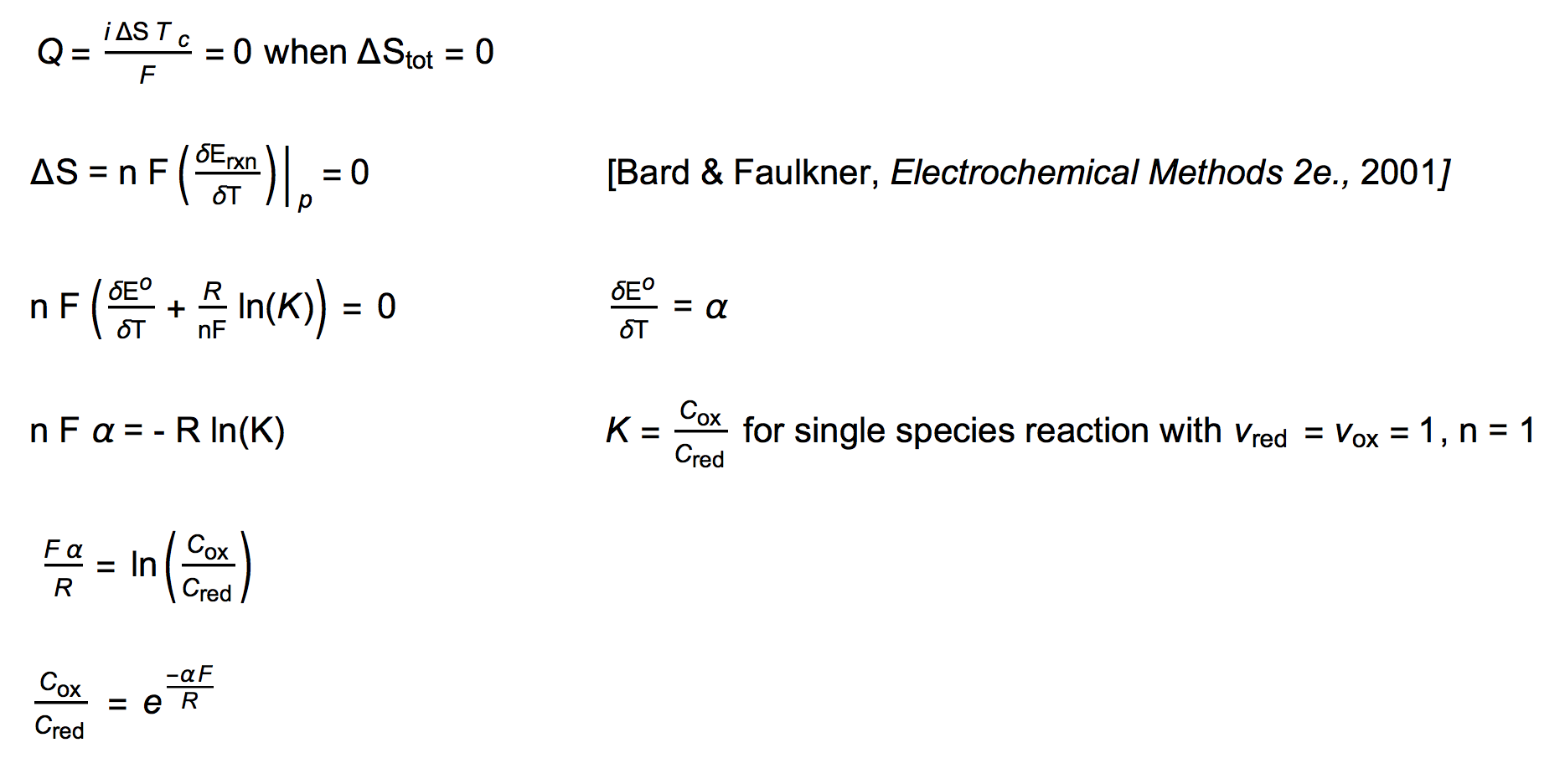


This concentration ratio indicates the extent of reaction past for which no cooling is thermodynamically possible. Practically, this ratio depends strongly on *α*. For example, ΔS = 0 for ferricyanide reduction at [Fe(CN)_6_^3-^]/[Fe(CN)_6_^4-^] = 3.6×10^7^ (α_FCN_=1.5mV/K) but at only [V^5+^]/[V^4+^] = 10 (α_V(IV/V)_= -0.2mV/K) for pervanadyl reduction.

**Comparing the scaling of limiting current density *I_lim_* and heat transfer coefficient *U* with flow velocity *v***

The derivation of the relationship between *I_lim_* and *v* is similar to a treatment presented by Dr. Martin Bazant at M.I.T. in 2013. For a fluid with reactant concentration *c* flowing at constant velocity over a reacting surface, the relevant mass transport equation is^1^:


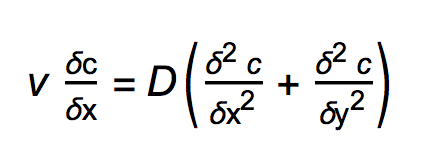


Where *x* is distance along the electrode in the direction of flow and *y* is the direction normal to the planar electrode. The expression can be simplified by the assumption that convection dominates diffusion in the direction of flow (electrochemical Peclet number *Pe*>>1).


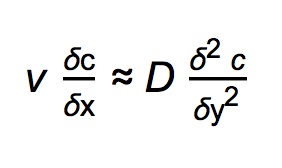


Estimating both terms relative the inlet electrolyte concentration *C_i_* and boundary layer thickness *z* further simplifies to:


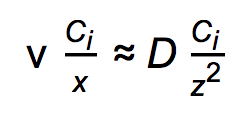


This expression yields an approximate boundary layer thickness of *z ≈ (Dx/v)^1/2^*. The reactant flux *B* diffusing across this boundary layer can similarly be approximated *B = D dc/dy ≈ DC_i_ /z*. Substituting the earlier expression for *z* gives an approximation for the reactant flux *B ≈ C_i_ (Dv/x)^1/2­^.* Integrating this expression over the total length *L* of the electrode yields an expression for the mass-flux limited current *I_lim_* that scales with *v^1/2^*.


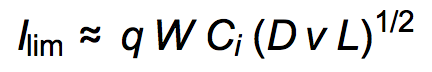


Where *W* is the electrode width and *q* is the charge passed per reactant molecule.

For heat transfer from an isothermal flat plate to a developing laminar fluid flow with Prandtl number Pr >0.6, the following correlation is derived in many sources^2^.


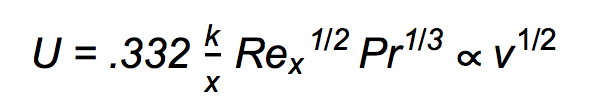


This represents the local heat transfer coefficient on the plate at location *x* in the direction of flow for a fluid with thermal conductivity *k*. *Pr* *≈ 5* for 800mM ferro/ferricyanide. Since the flow’s Reynold’s number *Re_x_* at location *x* is linearly proportional to flow velocity *v*, the heat transfer coefficient *U* is also expected to scale with *v^1/2^*.

**Calculating Q_g/b_ and ΔT_max_ for a variety of redox couples**

The specific heat *c_p_* and density *ρ* of the V^2+/3+^ and Fe(CN)_6_^3-/4-^ solutions and the thermopower *α* of the V^2+/3+^, V^4+/5+^, Fe(CN)_6_^3-/4-^ , Fe^2+/3+^and Br_2_/Br^-^ redox couples were measured as described previously^3^. For dissolved redox couples other than V^2+/3+^ and Fe(CN)_6_^3-/4-^, *c_p_* and *ρ* were used for NaCl solutions of the same total ionic strength^4^. For redox couples other than V^2+/3+^, V^4+/5+^, Fe(CN)_6_^3-/4-^ , Fe^2+/3+^and Br_2_/Br^-^, *α* was taken from calculations based on formation values^5^.

The activation loss *E_a_* was estimated based on literature sources for rotating disc electrode measurements of exchange current density *i_o_* or reaction velocity *k_o_*. Since *i_o_* and *k_o_* were generally not available at multiple temperatures as is required to establish a traditional activation energy, *E_a_* was instead approximated via the Butler-Volmer relation as the overpotential applied to pass 1mA/cm^2^ for the reduction (if *α*>0) or oxidation process (if *α*<0). Since a variety of *i_o_* values have been reported for each redox system, middle-of-the-road values were chosen. Higher values can be found for each couple, generally on noble metal catalysts. Significantly lower values can also be found reported as reaction velocities on Hg and Au, generally from older studies^6^. Notably, reaction velocities reported at low reactant concentrations were often inconsistent with exchange current densities established at higher concentrations. Where such discrepancies existed, the more frequently reported value was used.

**Ruling out an ionocaloric effect in the ferro/ferricyanide system**

Electrochemical cooling can also be a result of the change in entropy caused by capacitive ion rearrangement. As a result of this, temperature fluctuations up to about 0.5°C have been observed in some commercial supercapacitors^7,8^. In order to evaluate the cooling contribution induced by this capacitive effect rather than the Faradaic process in this study, we can model the capacitive cooling of an electric double layer capacitor (EDLC) of the same geometry and composition as the cell used here. An EDLC cooling cycle can be modeled as a heat engine with two fast isentropic steps—when the potential is applied and removed across the electrodes, resulting in ion rearrangement and changes in the system temperature—separated by two isoelectric steps, in which heat is exchanged with the environment. The conservation of energy for this process can be described by $TdS=C_{heat}dT+EdP$, where $T$, $S$, $C_{heat}$, and $P$ are the system’s temperature, entropy, heat capacity, and electric polarization respectively, and $E$ is an applied external electric field.

The maximum temperature change for a perfect cycle can be predicted by $TdS=C_{heat}dT$, or $\ln\frac{T_{1}}{T_{4}}=\frac{S_{1}-S_{4}}{C_{heat}}$ after integration, where state 1 refers to the system before application of the electric field, and state 4 refers to the system after removal of the electric field. The actual temperature difference will be reduced as a result of Joule heating, which can be calculated by the following correction to $T_{1}$: $T_{1}^{'}=T_{1}+\frac{I^{2}Rt}{C_{heat}}$, where $I$ is the capacitive current, and $R$ is the resistance. This Joule heating term assumes a constant-current discharge rate, chosen such that it maximizes the net cooling heat flux. The net cooling effect is the cooling heat flux minus Joule heating:  $\dot{Q}_{cool,net}=\dot{Q}_{cool}-\dot{Q}_{JH}=\Pi I-I^{2}R$. Here $\Pi$ is analogous to a Peltier coefficient, $\Pi=\frac{T\Delta S}{Nq}$.

The change in entropy that drives the change in temperature corresponds to counterions collecting at the electrode surfaces upon application of a potential. The configurational entropy of these counterions can be described by $\Delta S=-N_{counter}k_{B}\ln\left( \frac{V^{*}}{V} \right)$, where $N_{counter}$ is the number of counterions that migrate to the electrode surfaces to oppose the applied potential, $k_{B}$ is the Boltzmann constant, $V^{*}$ is the volume occupied by these counterions after migration to the surface, and $V$ is the total volume of electrolyte^7,9–11^. The number of counterions that collect at the surface is approximated from the cell capacitance, $C$ (modeled as a parallel plate capacitor in the Stern layer in series with a Gouy-Chapman capacitor in the diffuse layer, with the overall capacitance driven by the diffuse layer capacitance^12–18^), half-cell potential, $U_{half-cell}$, and counterion charge, $q_{counter}$: $N_{counter}=\frac{CU_{half-cell}}{q_{counter}}$. While not all ions that migrate to the electrode surface will be fully desolvated, this effect should be offset by the fact that more ions than approximated here will move as a result of the layering of counterions and co-ions^9^. At larger potentials, the rearrangement of the co-ions and solvent molecules, $N_{co/solv}$—being replaced by counterions at the electrode surface—becomes increasingly important, and the entropy change can be approximated using a lattice model of mixing: $\Delta S=-k_{B}\left( N_{counter}\ln\left( \frac{V^{*}}{V} \right)+N_{co/solv}\ln\left( \frac{V-V^{*}}{V} \right) \right)$. Note that this calculated entropy change is conservatively large, assuming all charge is countered without any Faradaic process, thereby maximizing the potential capacitive cooling contribution.

The resulting predicted capacitive temperature change for the cell used in this system is plotted for the range of relevant applied cell potentials in Figure S6. The cell parameters used for this calculation are summarized in Table S1. The observed cooling, on the order of 10^-1^ °C, goes far beyond what capacitive cooling alone would predict, on the order of 10^-5^ °C in cooling, indicating that the cooling process reported in this work is predominately Faradaic.

**Further information on the infrared electrode temperature measurement**

The measured emissivity of the carbon paper electrodes used in this study was 0.98. The thermal microscope (Infrascope TM by Quantum Focus Instruments) claims an absolute error of less than 2% of the deviation from the calibration temperature for this emissivity and a resolution as low as 1μK using A.C. techniques.

When used with carbon paper electrodes immersed in flowing electrolyte, the IR thermography data in this study had an overall noise level (standard error) of 10mK. Lower noise levels were obtained with Pt-black coated graphite felt electrodes (Figure S2), however, these were not used in the refrigeration study because the catalyst coating was suspected of detaching from the electrodes with 1mm/s flow. Additionally, the spatially-averaged temperature trace obtained for each electrode was observed to depend slightly on a subjectively chosen region of integration. Regions of integration were kept identical for successive tests in each series and error bars are included as the standard error of the temperature measurement. In addition to the challenge in providing confirmatory measurements with an auxiliary system (i.e. parylene-coated micro-thermocouples in contact with the electrodes), these difficulties mean that the reported temperature deviations and inferred refrigeration power in this paper should be regarded as estimates. We suggest that carefully calibrated point temperature measurement of the flowing electrolyte or even fully differential calorimetry might be the best route to quantitative assessment and even optimization of this and similar Faradaic refrigeration schemes.

*
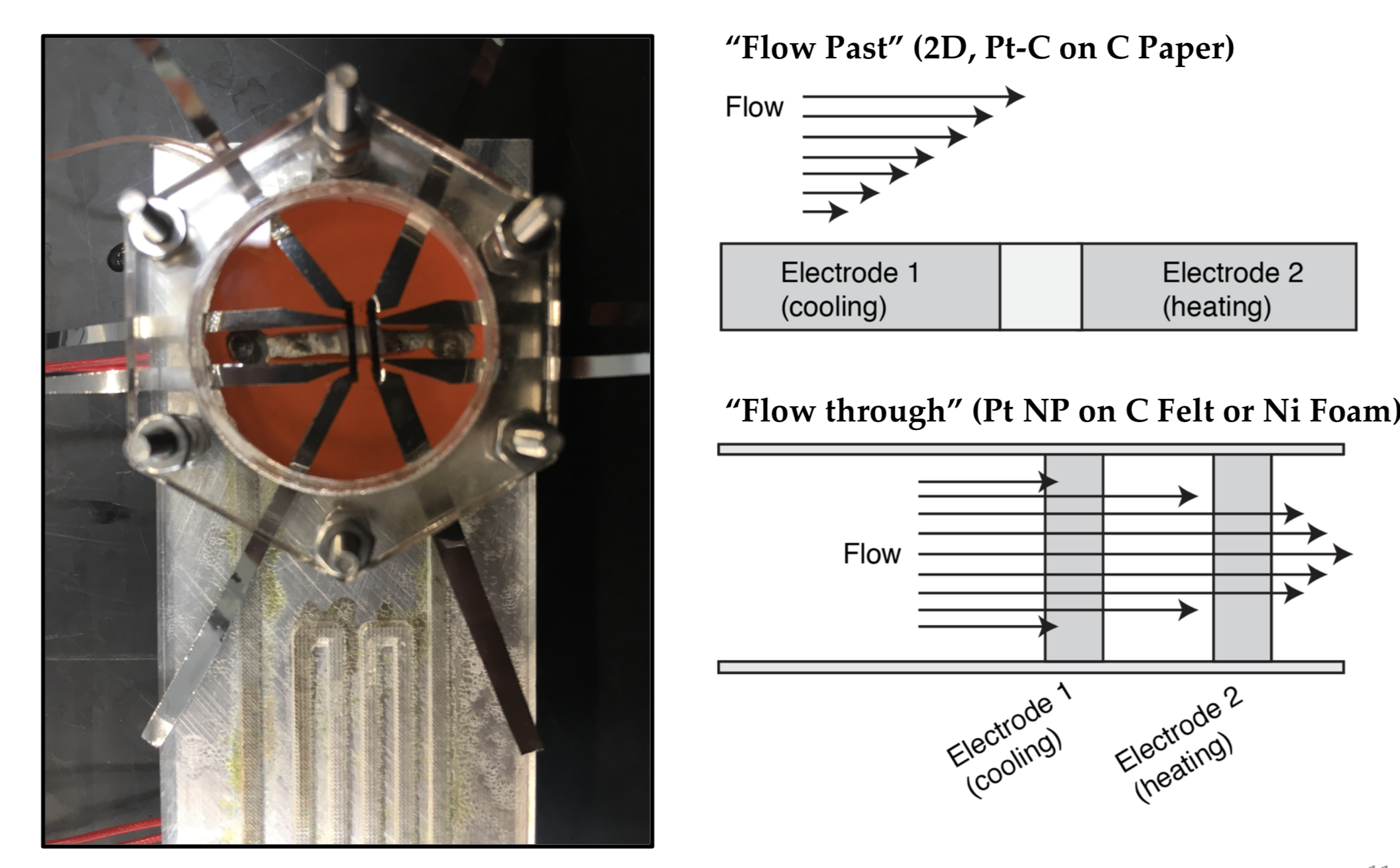
*

**Figure S1: Flow cell geometry viewed through CaF_2_ window.**

**a)**
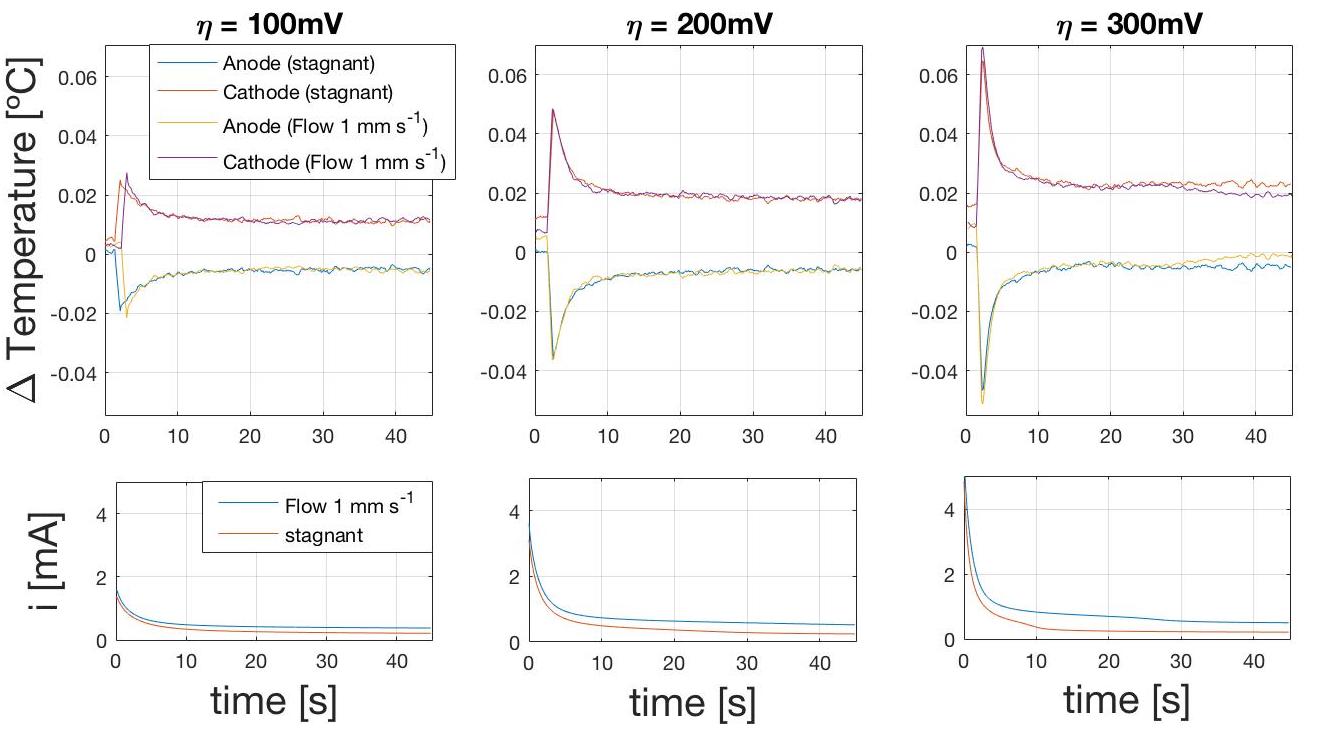
 **b)
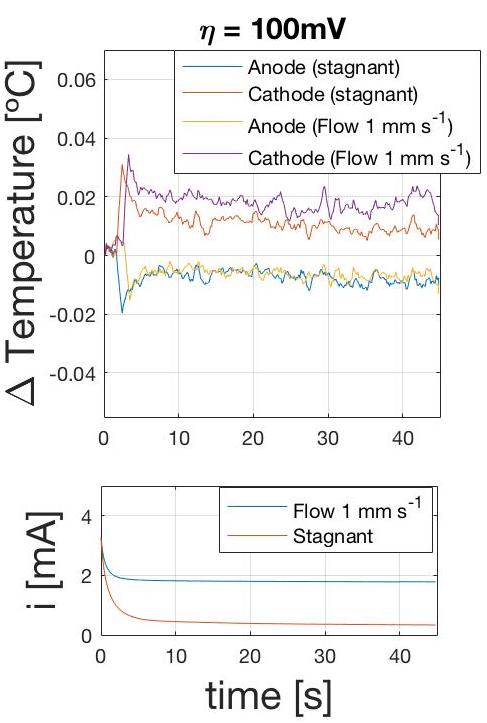
**

**Figure S2: Temperature profiles related to current density at two electrolyte flow rates and with two different catalyst loadings on ~20mm^3^ graphite felt flow-through electrodes.** As seen in other figures, additional cooling power as a result of higher current with higher flow rate is compensated by higher heat removal by electrolyte. S2a) shows a high catalyst loading (~300mg/cm^3^), while S2b) shows a lower catalyst loading (~30mg/cm^3^). The higher catalyst loading allowed lower noise level IR thermography, but clearly hindered electrolyte flow and led to lower overall reaction rates.

*
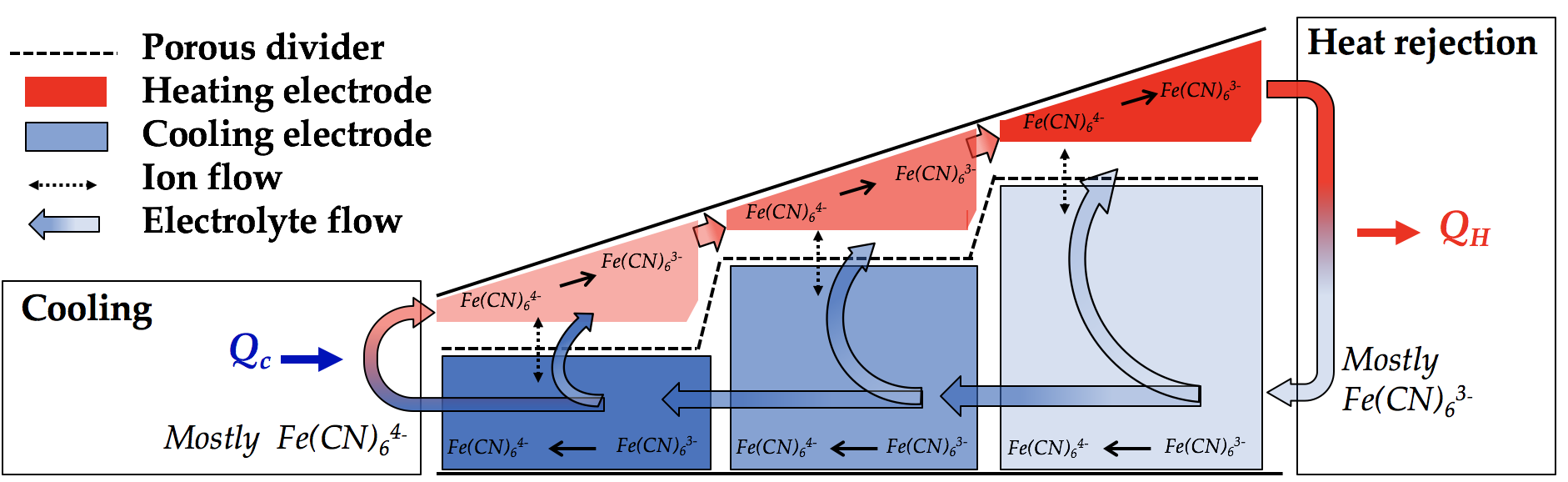
*

**Figure S3: Possible implementation using tapered flow-through electrodes for larger ΔT**. In this comfiguration, electrolyte flow pushes joule-heated electrolyte away from multiple cooling junctions in series. This configuration achieves a partial decoupling of thermal and ionic transport lengths.

*
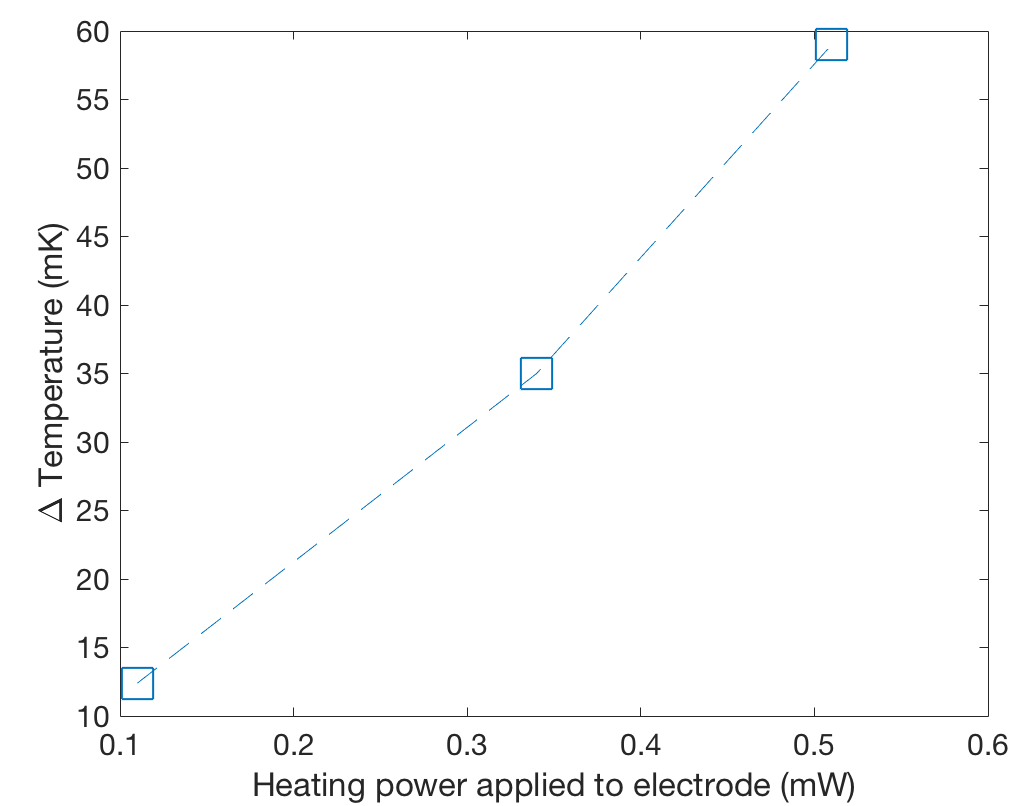
*

**Figure S4: Establishing the convective heat transfer coefficient for the flow cell anode with 1mm/s electrolyte flow***.*


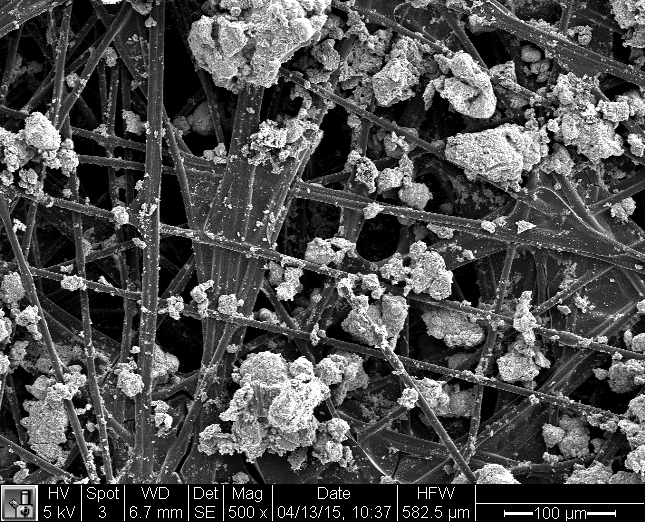

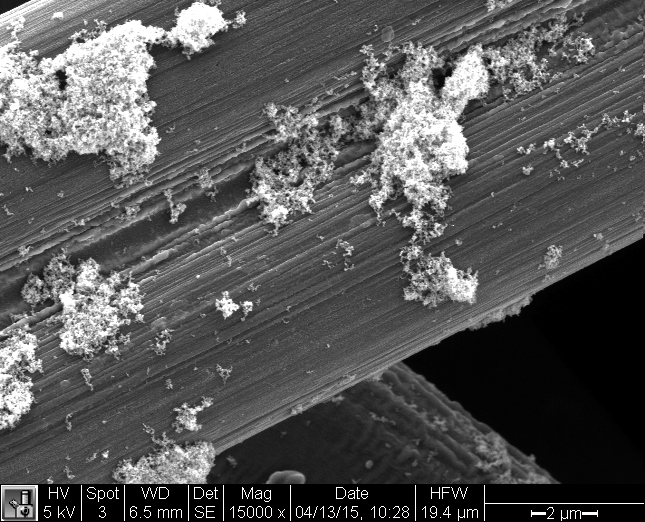


**Figure S5: SEM image of the porous cooling electrodes as prepared with Pt/C catalyst.**

*.*


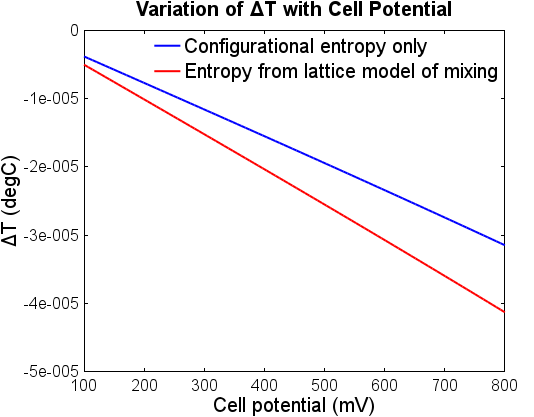


**Figure S6: Anticipated capacitive contribution to the temperature depression in the electrochemical cell.**


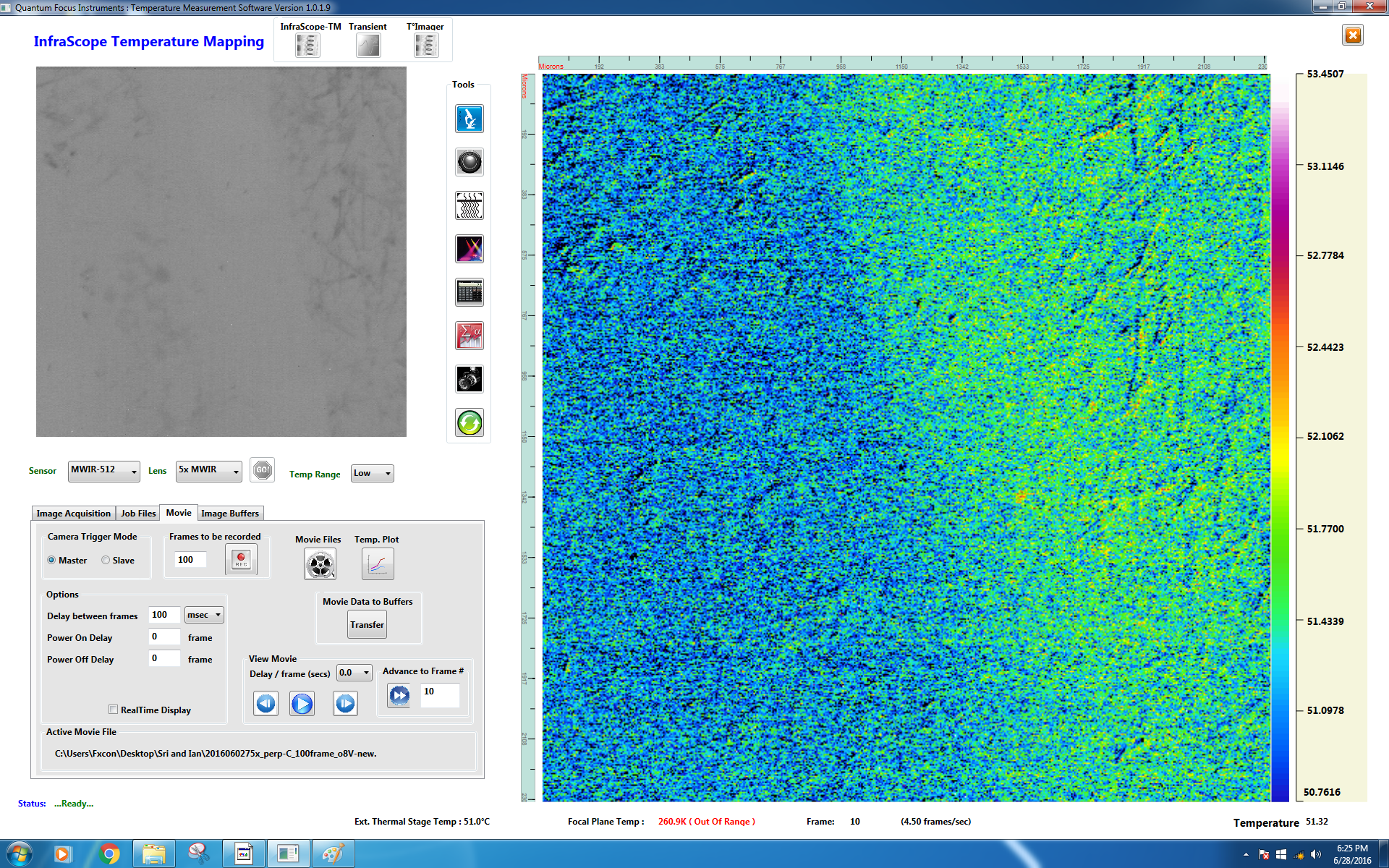


**Figure S7: Visualization of the cooling process in QFI software.**

**Table S1**

Temperature: 50ºC

Cell potential: varied from 100mW to 800mV

Separator thickness: 1mm

Heat capacity of separator: 0 (chosen conservatively to maximize cooling potential)

Density of separator: 0

**Electrolyte**

Thickness of separator + electrolyte: 1mm

Electrolyte concentration: 800 mM

Ion charge: 1.275 - the average ion charge (positive or negative) based on a mixture of K_3_Fe(CN)_6_/K_4_Fe(CN)_6_

Number of cations per K_3_Fe(CN)_6_ or K_4_Fe(CN)_6_: 3.5

Number of anions per K_3_Fe(CN)_6_ or K_4_Fe(CN)_6_: 1

Ion mobility: 8×10^-8^ m^2^/V/s

Heat capacity of electrolyte: 3500 J/kg/K

Density of electrolyte solution: 1100 kg/m^3^

Molar mass of solvent: 18.02×10^-3^ kg/mol

Stern layer thickness: 0.4 nm

Relative permittivity of Stern layer: 2.23

**Electrode**

Electrode thickness: 100 μm

Area of electrode: 100μm x 500μm

Heat capacity of electrode: 3500 J/kg/K

Density of electrode: 1000 kg/m^3^

Surface area per gram of electrode: 1000 m^2^/g

1. Bard, A. J. & Faulkner, L. R. *Electrochemical methods: fundamentals and applications*. (Wiley, 2001).

2. Stetsyuk, V., Kubiak, K. J., Liu, L. & Chai, J. C. An alternative approach to evaluate the average Nusselt number for mixed boundary layer conditions in parallel flow over an isothermal flat plate. *Int. J. Mech. Eng. Educ.* **46**, 241–251 (2018).

3. Poletayev, A. D., McKay, I. S., Chueh, W. C. & Majumdar, A. Continuous electrochemical heat engines. *Energy Environ. Sci.* **11**, 2964–2971 (2018).

4. Lide, D. *Handbook of Chemistry and Physics*. (CRC Press, 2012).

5. Bratsch, S. G. Standard Electrode Potentials and Temperature Coefficients in Water at 298.15K. *J. Phys. Chem. Ref. Data* **18**, 1–21 (1989).

6. Weber, A. Z. *et al.* Redox flow batteries: A review. *J. Appl. Electrochem.* **41**, 1137–1164 (2011).

7. Gualous, H., Louahlia, H. & Gallay, R. Supercapacitor Characterization and Thermal Modelling With Reversible and Irreversible Heat Effect. *IEEE Trans. Power Electron.* **26**, 3402–3409 (2011).

8. Schiffer, J., Linzen, D. & Sauer, D. U. Heat generation in double layer capacitors. *J. Power Sources* **160**, 765–772 (2006).

9. Bazant, M. Z., Storey, B. D. & Kornyshev, A. a. Double layer in ionic liquids: Overscreening versus crowding. *Phys. Rev. Lett.* **106**, 6–9 (2011).

10. Grunwald, E. & Steel, C. Solvent Reorganization and Thermodynamic Enthalpy-Entropy Compensation. *J. Am. Chem. Soc.* **117**, 5687–5692 (1995).

11. Zheng, J. P. & Jow, T. R. Effect of of Salt Concentration in Electrolytes on the Maximum Energy Storage for Double Layer Capacitors. *J. Electrochem. Soc.* **144**, 2417–2420 (1997).

12. Bonnefont, A., Argoul, F. & Bazant, M. Z. Analysis of diffuse-layer effects on time-dependent interfacial kinetics. *J. Electroanal. Chem.* **500**, 52–61 (2001).

13. Brown, M. A., Goel, A. & Abbas, Z. Effect of Electrolyte Concentration on the Stern Layer Thickness at a Charged Interface. *Angew Chem Int Ed* 5 (2016).

14. Chmiola, J., Largeot, C., Taberna, P.-L., Simon, P. & Gogotsi, Y. Desolvation of Ions in Subnanometer Pores and Its Effect on Capacitance and Double-Layer Theory. *Angew. Chem.* **120**, 3440–3443 (2008).

15. Huang, J., Sumpter, B. G. & Meunier, V. A Universal Model for Nanoporous Carbon Supercapacitors Applicable to Diverse Pore Regimes, Carbon Materials, and Electrolytes. *Chem. - Eur. J.* **14**, 6614–6626 (2008).

16. Prehal, C. *et al.* Tracking the structural arrangement of ions in carbon supercapacitor nanopores using in situ small-angle X-ray scattering. *Energy Environ. Sci.* **8**, 1725–1735 (2015).

17. Reszko-Zygmunt, J., Sokołowski, S., Henderson, D. & Boda, D. Temperature dependence of the double layer capacitance for the restricted primitive model of an electrolyte solution from a density functional approach. *J. Chem. Phys.* **122**, 084504 (2005).

18. Wang, H. & Pilon, L. Accurate Simulations of Electric Double Layer Capacitance of Ultramicroelectrodes. *J. Phys. Chem. C* **115**, 16711–16719 (2011).
